# Glial Ion Channels in Myelin Pathophysiology: Insights from Leukodystrophies

**DOI:** 10.3390/life15121922

**Published:** 2025-12-15

**Authors:** Marcello Belfiore, Sergio Visentin, Elena Ambrosini

**Affiliations:** 1 National Center for Preclinical and Clinical Evaluation and Research of Drugs, Istituto Superiore di Sanità, Viale Regina Elena, 299, 00161 Rome, Italy; marcello.belfiore@iss.it (M.B.); visentin.sr@gmail.com (S.V.); 2 Department of Neuroscience, Istituto Superiore di Sanità, Viale Regina Elena, 299, 00161 Rome, Italy

**Keywords:** oligodendrocytes, astrocytes, microglial cells, MLC1, TMEM63A, ClC-2

## Abstract

Leukodystrophies (LDs) constitute a heterogeneous group of genetic diseases in which mutations in glial cell genes lead to alterations in myelin formation and/or maintenance, ultimately causing white matter dysfunction. Increasing evidence on the genetic basis of LDs has revealed that proteins expressed not only by myelin-forming oligodendrocytes, but also by other glial cell types, play essential roles in myelination. By elucidating disease mechanisms, these studies have uncovered novel cellular and molecular contributors to myelin biogenesis and function, including ion channels. This is exemplified by the recent identification of mutations in the *TMEM63A* gene, which encodes the homonymous mechanosensitive channel, as the causative factor of the rare hypomyelinating LD HLD19 and by mutations in the chloride channel *ClC-2* as responsible for the development of the vacuolating ClC2 LD. Together, this evidence has opened new perspectives on the crucial role of mechanosensitivity and ionic homeostasis for proper myelin development and structural integrity. In this review, we summarize recent advances on the role of glial ion channels in healthy white matter development and preservation, as well as their direct and indirect contributions to LD pathomechanisms. Finally, we discuss emerging therapeutic implications of these studies for LDs and other demyelinating conditions and emphasize the considerable potential of a cross-pathological, integrative approach to uncover shared and disease-specific mechanisms of demyelination.

## 1. Introduction

Leukodystrophies (LDs) include a heterogeneous group of rare genetic disorders characterized by the depletion or alteration of white matter (WM) of the central nervous system (CNS). The clinical course is mostly progressive and often fatal, but static or even improving forms are present. To date, approximately 50 different disease entities have been described, with an incidence ranging from 1 in 8000 to 1 in 100,000 live births, depending on the specific LD [1,2,3]. In these pathological conditions, magnetic resonance imaging (MRI) is the main tool for the first diagnosis, followed by metabolic testing and, where possible, biomarkers as guidance for targeted genetic tests. With advances in molecular diagnostics, particularly whole-exome sequencing (WES) and whole-genome sequencing (WGS), the accuracy and timeliness of LD diagnosis have significantly improved, enabling the identification of disease-linked genes, and greatly increasing the number of newly discovered LDs [1,2,3].

Depending on the mutated gene and the function of the encoded protein in WM formation and maintenance, LDs may manifest from birth or in early childhood, although later-onset, milder forms also occur. Common symptoms include varying degrees of cognitive dysfunction and severe motor impairments, loss of ambulatory capacities, ataxia and spasticity, often associated with epilepsy, dystonia, peripheral neuropathy, macrocephaly or microcephaly. In some cases, the grey matter, the peripheral nervous system (PNS), or other organs are also involved [1,2,4].

Although no disease-modifying therapies are available for most LDs, new in vivo and ex vivo gene therapy approaches are emerging for a limited number, together with bone marrow or hematopoietic stem cell transplantation (HSCT), which have shown success in slowing disease progression [5,6]. Clinical trials evaluating small molecules, enzyme replacement, or antisense oligonucleotides are also ongoing for selected LDs [5,6].

Growing knowledge on LD molecular pathogenesis is opening new avenues for the identification of molecular targets for pharmacological approaches aimed at arresting or slowing disease progression [7].

Although the pathogenic mechanisms of LDs converge on WM abnormalities through decreased myelin formation (hypomyelinating LDs), loss of existing myelin (demyelinating LDs), defective myelin deposition (dysmyelinating LDs) or myelinolytic processes (vacuolization of myelin/cystic) [1,3,4], the genetic background of these disorders is highly heterogeneous.

Increasing genetic studies have shown that myelin defects in LDs may arise not only from mutations in genes encoding proteins directly involved in myelin formation, maintenance, or in the development and function of the myelin-forming oligodendrocyte cells but also from mutations occurring in genes encoding proteins expressed in other glial cells, particularly astrocytes, microglia, as well as in other CNS components such as axons or blood vessels [3,4,8].

These discoveries, together with subsequent mechanistic studies in animal and cellular models that revealed a variety of molecular and cellular defects, have substantially advanced our understanding of the complexity of the mechanisms underlying the biogenesis and maintenance of healthy WM [9].

In this context, the finding that dysfunctions in some ion channels, expressed by glial cells and involved in the translation of mechanical forces to ion fluxes and in the regulation of ionic and water homeostasis, contribute to LD pathogenesis has underscored the importance of these biological processes in myelin formation and function.

Indeed, mutations in the mechanosensitive ion channel TMEM63A have been recently linked to the hypomyelinating LD HLD19 [10], highlighting the role of mechanosensitivity in oligodendrocyte development and myelin formation.

Similarly, the LDs caused by mutations in the chloride channel ClC-2 revealed the significant contribution of the homeostatic control of chloride and water fluxes for the maintenance of WM structural integrity [11,12].

In this review, we will briefly introduce the myelination process and summarize the role of the main ion channels expressed by glial cells in the development and maintenance of healthy myelin, organizing their description according to the specific glial cell population that expresses them.

We will then describe current evidence of glial ion channels directly and indirectly involved in the pathological mechanisms of LDs, along with the underlying molecular pathways.

Finally, we will discuss the new insights derived from these findings and the therapeutic potential of ion channels as pharmacological targets for LDs, as well as their relevance to other demyelinating diseases.

## 2. Glial Cells

**Figure 1:**
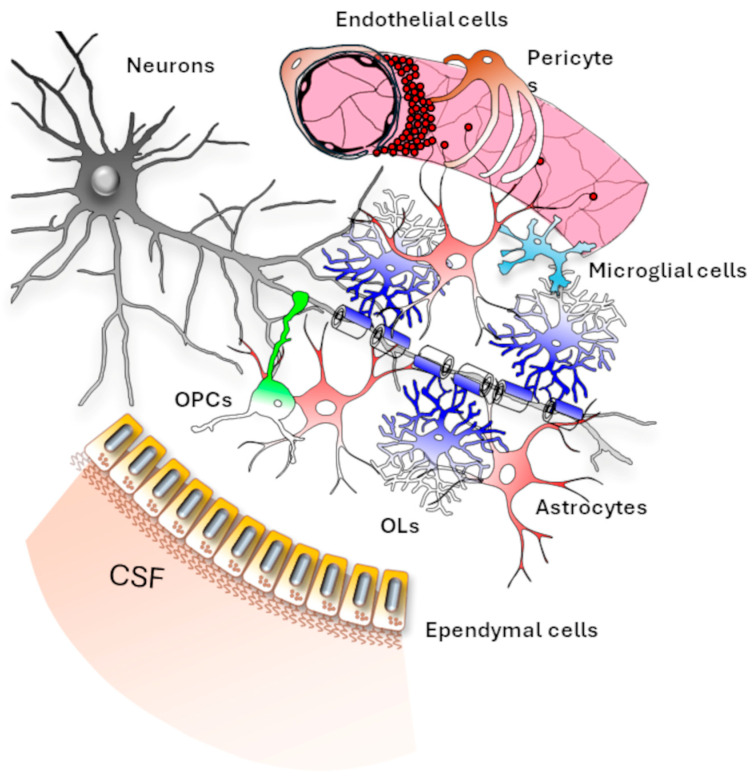
***Glial cell relationships in the central nervous system (CNS)***. CNS glial cells include several types, each with specific functions. Oligodendrocyte progenitor cells (OPCs, green) and oligodendrocytes (OLs, blue) are involved in the myelination and maintenance of axons. Astrocytes (red) support neuronal synaptic plasticity and axonal metabolism and maintain electrolyte balance and tissue ionic homeostasis. Additionally, astrocytes contribute to the formation of the blood–brain barrier and are key players of the neurovascular units, working alongside endothelial cells (pink) and pericytes (orange). Microglial cells (light blue) are resident macrophages in the CNS, able to trigger immune responses against non-self epitopes and to regulate inflammatory responses within the CNS. Ependymal cells (yellow) are epithelial-like cells that regulate the exchange of solutes and electrolytes in cerebrospinal fluid (CSF). Neurons are represented as grey cells.

Glial cells are essential components of the nervous system, performing a variety of crucial supportive and regulatory functions and acting as both protectors of tissue and organ health and potential contributors to disease pathogenesis [13,14]. They include oligodendrocytes (OLs) and their progenitors (oligodendrocyte progenitor cells, OPCs), astrocytes, and microglia in the CNS, as well as Schwann cells and satellite glial cells in the PNS. Each glial cell type fulfils specific roles in supporting neural development, brain tissue homeostasis, neuronal activity, and myelin formation and maintenance (Figure 1).

Astrocytes are multifunctional cells that, by contacting all the other cellular components of the CNS, are involved in maintaining electrolyte balance and tissue ionic homeostasis, regulating neurotransmitter uptake, and taking part in the formation and function of the blood–brain barrier (BBB) [14,15]. The BBB is a highly selective and dynamic interface that separates the circulating blood from the brain parenchyma, and that is primarily formed by endothelial cells, astrocytic endfeet, pericytes, and the basement membrane. Astrocytes also support neuronal metabolism and are fundamental components of the neurovascular unit (NVU), a functional structure that integrates neurons, glial cells, and vascular cells to regulate cerebral blood flow according to neuronal energy demands, thereby ensuring that neurons receive adequate oxygen and nutrients in response to their activity. They also contribute to tissue repair by becoming reactive after injury (astrogliosis), limiting damage spread, forming a protective glial scar, and secreting trophic factors that sustain neuronal survival and regeneration [14,15]. Thus, some LDs are related to astrocytes misfunctioning, as in the case of MLC and CLCN2-related leukoencephalopathy.

OPCs and OLs are responsible for the myelination of axons and thus for conduction velocity and axonal integrity. They also provide metabolic and trophic support to axons and help preserve ionic homeostasis in both axons and myelin, thereby contributing to the stability and integrity of neural circuits [13]. Therefore, their malfunctioning could lead to LD, as for the mutation of *TMEM63A*.

Microglia are the central nervous system’s resident immune cells that monitor the brain tissue for injury or infection, remove dead cells and debris, regulate inflammation, prune unnecessary synapses, and release factors that support neuron survival and repair [14,15]. Due to their capability of mediating inflammatory responses and synaptic plasticity, they are involved in LD pathogenesis.

Together with these glial cells, ependymal cells in the ventricular system complete the landscape of non-neuronal cells in the CNS. These epithelial-like cells line the ventricles and are essential for the production and circulation of cerebrospinal fluid (CSF), as well as for the exchange of substances between the CSF and brain tissue [15,16]. From this point onward, we will focus on CNS processes and components. For detailed descriptions of PNS-related cellular composition and molecular mechanisms of myelin formation, readers are referred to specific reviews [17].

## 3. Myelin Formation

Myelination is a dynamic and highly regulated process where a specialized membrane wraps around axons, providing electrical insulation and constraining sodium channels, responsible for the action potential initiation and propagation, to the unmyelinated gaps between myelin segments (nodes of Ranvier) and potassium channels beneath the myelin sheath [18]. This enables rapid and energy-efficient nerve impulse conduction for saltatory propagation. In the CNS, myelin is formed by OLs that ensheathe neurons with their membrane extensions, wrapping axons in multiple layers. Each oligodendrocyte can wrap around up to 50 different axons [19,20].

### 3.1. The Myelination Process

The entire myelination process consists of several critical steps, most of which are defective in different types of LDs, including: (1) proliferation of OPCs; (2) recognition of axons; (3) differentiation of OPCs into myelinating OLs; (4) membrane outgrowth and axonal wrapping; (5) transportation of membrane components; and (6) myelin compaction [21]. Following glial cell lineage specification, OPCs rapidly proliferate and migrate throughout the CNS in response to various external signals, including axon-associated factors such as Polysialyl Acid Neural Cell Adhesion Molecule (PSA-NCAM), Neuregulin-1, and laminin-α2 [22,23]. Axon diameter also plays a key role: typically, only axons with a diameter greater than 0.2 µm are myelinated [24]. Once OPCs establish contact with axons, they differentiate into mature OLs and begin producing large amounts of lipids and myelin proteins to form the myelin sheath.

Whatever misfunctioning of this multistep process might result in LD. In the following sections, we will discuss the mechanisms that may impair physiological functions.

### 3.2. Myelin Composition

Myelin is thus a modified cell membrane that wraps multiple times, up to 100 layers, around the nerve axons. It is primarily composed of lipids (70–85%) and proteins (15–30%), with a minor water content. Key lipids include cholesterol (about 40% of the total lipid content), phospholipids (40%, mainly phosphatidylcholine, sphingomyelin, and plasmalogen), and glycolipids (20%, mainly galactocerebrosides and galactosulfatides). In the outer leaflet, lipid rafts, which are small membrane microdomains enriched in cholesterol and sphingolipids, enhance myelin stability. Under electron microscopy, two lines are visible in the myelin structure: the major dense line (MDL), the dark line formed by the condensed intracellular cytoplasmic component between the inner membranes of the two lipid bilayers (2–3 nm wide), and the lighter intraperiod line, formed by the apposition of the outer (extracellular) sides of the oligodendrocyte membranes (about 4 nm wide) [25,26,27,28]. The main myelin protein components are the proteolipidic protein (PLP) and the myelin basic protein (MBP). PLP is the most abundant myelin protein in the CNS, constituting 38% of the total myelin protein mass. This integral membrane protein plays a fundamental role in compacting the multilayer membrane structure, particularly at the intraperiod line. PLP molecules on opposing membrane layers interact with each other via homophilic binding across the extracellular space to stabilize the close apposition of the adjacent membrane layers, thus contributing to tight membrane stacking [26,28,29]. Mutations in the *PLP1* gene may result in hypomyelinating LD, like the Pelizaeus-Merzbacher disease (PMD) [30]. The MBP is the second most abundant myelin protein in the CNS, constituting about 30% of dry protein mass in the myelin. Being a positively charged hydrophobic protein, it binds electrostatically to negatively charged lipids on the inner membrane surface, stabilizing the compact structure at the major dense line [31,32,33].

### 3.3. Myelination Progression During Development

During human nervous system development, myelin production begins in the last third of embryonic development and peaks postnatally. By the second year of age, the majority of myelination is complete, as demonstrated by MRI, even if in some CNS regions the process is not completed until early adulthood [34]. Both intrinsic genetic programs and extrinsic signals, derived not only from oligodendrocytes but also from the other cellular components of the brain parenchyma, such as OPCs, astrocytes, and microglia, take part in this complex process. This involves transcriptional and post-transcriptional mechanisms, secreted molecules, as well as neuronal activity in a bidirectional communication system between glial cells and neurons [35,36,37]. It has also been recently found that the mechanical properties of the cellular environment play a critical role in the development and maturation of myelin-forming cells.

Ion channels play fundamental roles in glial functions, particularly by regulating intracellular calcium dynamics and shaping the electrical properties of myelinating cells. During the myelin formation process, they are crucial in guiding the progression of the oligodendrocyte lineage, myelin assembly, and long-term maintenance [38]. 

## 4. Role of Glial Ion Channels in Myelin Formation and Maintenance

### 4.1. Oligodendroglial Ion Channels in Myelination: Emerging Role for Oligodendrocyte Progenitor Cells (OPCs)

New evidence suggests that during brain development, OPCs establish functional synaptic-like contacts with neurons, allowing them to sense neuronal activity, which substantially guides OPCs in proliferation and differentiation towards mature myelinating OLs [33]. In the early stage of this development, OPCs express a very complex repertoire of ion channels, most of which they lose in later phases.

Families of OPCs ion channels and their roles include the following: (1) *Voltage-gated K^+^-channels* as Kv1.3-5, which allow for OPCs division and K^+^-buffering consequent to neuronal activity, while the K^+^-inward rectifiers, such as Kir 4.1 channels, drive OPC differentiation and myelination. Kir4.1 is a critical indicator of lineage progression due\to its crucial role in establishing the membrane resting potential. Worth noting, Kir4.1 knock-out (KO) in mice causes impaired OPC differentiation, defective myelination associated with myelin vacuolation [39,40]; (2) *Voltage-gated Na^+^-channels*, such as Nav1.2, that promote OPC maturation. Tetrodotoxin (TTX)-sensitive Nav1.2 channels guide axon-oligodendrocyte interactions and their selective KO results in impaired myelin sheath formation [41]; (3) *L-type Ca^2+^-channels* like Cav 1.2-3 are involved in oligodendrocyte maturation and myelination, as demonstrated by Cav1.2 conditional KO mice, which show a decrease in the percentage of myelinated axons associated with a reduction in the number of myelinating OLs and OPC proliferation [42]; (4) *different types of Cl^−^-channels* that, by contributing to ion homeostasis, volume regulation, signalling, and cell differentiation, exert critical roles for OPC and OL development and function [43]. They belong to different subgroups, which include: (4_a_) *voltage-gated chloride ClC-2 channels*, that function as positive regulators of OPC differentiation and influence myelin sheath integrity [44,45] as demonstrated by ClC-2 KO mice showing WM vacuolation and altered myelin topology (see below) [45]; (4_b_) *Bestrophins* (BEST1), which are calcium-activated anion channels permeable to Cl^−^ and HCO_3_^−^ expressed in OLs and astrocytes [46,47,48]. They regulate ion balance, pH, and cell volume. Moreover, they buffer Ca^2+^ signals, influence the OPC resting potential and release paracrine gliotransmitters (gamma-aminobutyric acid and glutamate) in a Ca^2^-dependent manner to promote OPC differentiation [46,49,50] and (4_c_) *TMEM16A*, belonging to the Anoctamins family of Ca^2+^-activated chloride channels. In the early developing CNS, the TMEM16A (ANO1) mediates the process extension of radial glial cells and the organization of the cortical layers [51].

In addition, the neuron-OPC interactions are also mediated by: (5) *neurotransmitter receptors* such as the α-amino-3-hydroxy-5-methyl-4-isoxazolepropionic acid receptors (AMPAR); the N-methyl-D-aspartate receptors (NMDAR), the Gamma-Aminobutyric Acid Receptors (GABA_A_R, GABA_B_R), and the purinergic ionotropic receptors (P2XR), (6) *glutamate transporters* Excitatory Amino Acid Transporter 1 (EAAT1/GLAST) and 2 (EAAT2/GLT-1), and (7) *ion exchangers* such as the sodium-calcium exchangers NCX1 and NCX3, which are all expressed only in immature OPCs and progressively lost in mature OLs [52].

Notably, GABARs play a crucial role in OPC maturation. OPCs widely express GABARs [53,54,55] as well as the enzymes for GABA synthesis, glutamic acid decarboxylase-67 and monoamine oxidase-B, enabling them to sustain maturation through autocrine and paracrine stimulation of neighbouring OPCs. The expression of these enzymes decreases during OPC differentiation [52]. In addition, GABA released by local interneurons influences OPC proliferation and maturation via both ionotropic GABA_A_Rs and metabotropic GABA_B_Rs [53].

GABA_A_Rs are ligand-gated ion channels permeable to Cl^−^ and HCO_3_^−^. Unlike in mature neurons, GABA induces membrane depolarization in OPCs, which activates voltage-gated calcium channels (VGCCs). The resulting rise in intracellular calcium promotes OPC proliferation and, through cAMP-dependent activation of CREB, drives the expression of MBP, thereby facilitating OPC differentiation [53,56]. GABA_B_Rs, in contrast, are G-protein-coupled receptors that can modulate VGCCs and, depending on context, adenylyl cyclase activity [57,58], as well as activate Fyn kinase [59]. These signalling pathways lead to cytoskeletal rearrangements and MBP expression, further supporting OPC differentiation and myelination [53,56].

During postnatal CNS maturation, OPCs constitute a self-renewing, heterogeneous population capable of differentiating into new myelinating OLs when needed; thus, in the postnatal brain, OPCs and mature OLs can cohabitate [52]. These precursors continuously detect and integrate neuronal signals that regulate their development and differentiation, making them essential for adaptive myelination [60,61]. OPCs extend elaborate cellular processes that receive direct input from neighbouring unmyelinated axons through synaptic-like contacts with both glutamatergic and GABAergic interneurons. The vesicular release of glutamate from axons stimulates OPC maturation and promotes the formation of myelin sheaths [52,62]. This process is mediated by calcium transients within the OPCs at the synaptic-like contacts, with Ca^2+^ entering from the extracellular medium or being released from intracellular stores. These calcium dynamics are driven by the direct activation of a variety of ion channels, ionotropic receptors, and ion exchangers located on OPC, including Cav 1.3 channels, AMPAR, and Na^+^/Ca^2+^ exchangers. Besides the contribution of Ca^2+^ influx through AMPA and P2X7 receptors, intracellular calcium spikes also arise from a complex mechanism of induction triggered by cell depolarization due to GABA-activation, Na^+^ entrance through Na_V_1.2 channels, and exchange with Ca^2+^ by the NCX exchanger [52,63].

Overall, these studies indicate that OPC proliferation and progression toward mature OLs are significantly regulated by dynamic changes in multiple ion channels mediating intimate OPC–neuron communication.

### 4.2. Ion Channels in Mature Oligodendrocytes

As OPCs differentiate into mature OLs and initiate axon myelination, they undergo a substantial shift in the expression of ion channels and transporters. In doing so, they acquire a repertoire of channels specialized in supporting axonal metabolism and maintaining ion homeostasis, ultimately influencing conduction efficiency.

**Figure 2:**
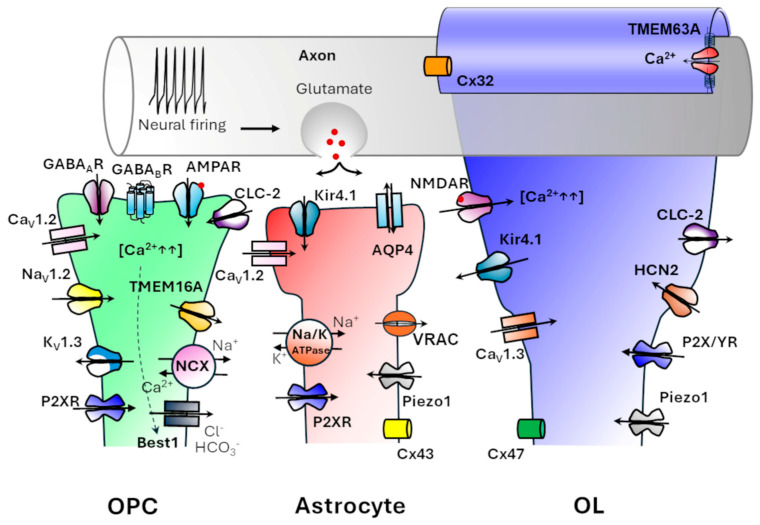
***Ion channel dynamics guiding oligodendrocyte progenitor cell (OPC) maturation toward myelinating oligodendrocytes (OLs)***. During postnatal brain development, OPCs form functional synaptic-like contacts with neurons, allowing them to sense neuronal activity and regulate their proliferation and differentiation into myelinating OLs. Immature OPCs express a broad repertoire of voltage-gated ion channels (Kv1.3, Nav1.2, Cav1.2, ClC-2, BEST1, TMEM16A/F), neurotransmitter receptors (AMPAR, GABA_A_R, GABA_B_R, P2XR) and ion exchangers (NCX1/3), most of which are downregulated upon differentiation. GABAergic autocrine and paracrine signalling through depolarizing ionotropic GABA_A_Rs and metabotropic GABA_B_Rs modulates intracellular Ca^2+^, promoting MBP expression and OPC differentiation. Mature OLs, in contrast, increase expression of Kir4.1 and ClC-2 channels and of other proteins necessary to support myelin sheath elongation, membrane tension, electrolyte balance, such as HCN2, Cav1.3, anoctamins, TMEM63A, while Nav and Kv channels disappear. Within myelin sheaths, Cx32-homotypic gap junctions between myelin lamellae, and heterotypic Cx43-Cx47 gap junctions between astrocytes and myelinating OLs establish glial network communication, allowing metabolic coupling with axons. In mature OLs, ionotropic NMDARs prevail over AMPARs, reflecting a shift toward activity-dependent metabolic support to myelin.

In particular, NMDARs increasingly predominate AMPARs in mediating synaptic-like input (Figure 2). The recruitment and activation of NMDARs facilitate the targeted mobilization of glucose transporter 1 (GLUT-1) into the myelin compartment, allowing glucose uptake to occur in an activity-dependent manner and driving enhanced glycolysis within OLs. The lactate and pyruvate generated through this process are then exported to the axons via monocarboxylic acid transporters (MCT-1 and MCT-2), so sustaining intense firing that, in turn, supports efficient myelination [52]. Indeed, due to the axonal insulation provided by myelin sheaths, the neuronal uptake of extracellular metabolites is limited by physical barriers [64]. Rinholm and colleagues demonstrated that in slice cultures maintained under low-glucose conditions, oligodendrocyte myelination was impaired; however, this impairment could be rescued by supplying exogenous lactate [65]. Furthermore, a conditional KO mouse model, lacking MCT1, resulted in late-onset hypomyelination and axonal degeneration [66]. Lactate is also crucial during development and remyelination, as it is needed to produce ATP and lipids essential for myelin maintenance [67].

In contrast to OPCs, which express voltage-gated Na^+^ channels, AMPARs, and various Kv channels that sustain excitability and proliferation, mature OLs predominantly express Kir4.1 [68,69] and ClC-2 [35,44,45], which are critical for K^+^ buffering and chloride and water homeostasis in the WM [68,69]. Moreover, during OL differentiation, the expression of voltage-gated calcium channels Cav1.3 rises, while the expression of Cav1.2 decreases. This shift promotes specific Ca^2+^-dependent signalling pathways that are essential for process extension, membrane wrapping, and activity-dependent myelination [70]. In mature OLs, GABARs expression also decreases, and their role changes from mediating fast synaptic inputs, as seen in OPCs, to modulating local network activity through activity-dependent, non-spiking mechanisms. Indeed, while OPCs are excitable and respond robustly to synaptic input, mature OLs have largely lost electrical excitability [62].

Hyperpolarization-activated, cyclic nucleotide-gated (HCN) ion channels, first characterized in cardiac pacemaker cells, where they are responsible for the “funny” current [71,72], are also involved in the control of myelination. These channels are expressed in both neurons [71,72,73,74] and glia [72,74] in the CNS. Recently, by combining electrophysiology and cellular biology techniques, Swire and colleagues revealed the expression of HCN2 channels in mature OLs and demonstrated their role in the regulation of myelin sheath length [75]. HCN2 channels are six transmembrane domains voltage-gated channels allowing Na^+^ and K^+^ ions to flow at membrane potentials more negative than −70 mV. Their cytoplasmic endings contain a cyclic nucleotide-binding domain (CNBD) controlling the gating. cAMP/cGMP acts as a positive modulator, shifting activation toward more depolarized potentials and increasing channel open probability [75,76]. The same authors, using experimental models both in vitro (CC1/SOX10-positive OLs) and in vivo (mice with conditional deletion of HCN2 in newly differentiated OLs), proved that depolarizing currents through HCN2 channels critically regulate the expression of key myelin proteins, affecting myelin sheath elongation but not the number of myelin sheaths. Collectively, these findings identify HCN2 channels as central regulators of myelin physiology.

OLs also express mechanosensitive cation channels, such as Piezo1 [77,78,79], and TMEM63A [10,35,80,81,82], which transduce mechanical signals from the extracellular matrix and vascular dynamics to myelination.

Piezo1 is a non-selective cation channel permeable to Ca^2+^, but also to Na^+^ and K^+^, which is mainly activated by mechanical stimuli such as membrane tension and tissue stiffness. In different types of cells, the downstream signalling effect of Piezo1 activation is the release of ATP and activation of ionotropic P2X and metabotropic P2Y purinergic receptors [77,83]. Although Piezo1 is mainly expressed in neurons, where it takes part in axon pathfinding, its activation in OLs drives nuclear translocation of the transcriptional activators YAP/TAZ, affecting OL proliferation and differentiation [77]. In general, Piezo1 activation exerts a negative role in CNS myelination, and it has been proposed as a possible pharmacological target for demyelinating diseases [84,85,86]. Indeed, in a murine organotypic cerebellar slice model, a Piezo1 antagonist (GsMTx4) has been found to attenuate demyelination induced by the cytotoxic lipid psychosine [86]. Similarly, Piezo1 inhibitors reduce the MBP degradation in a mouse model of demyelination following intracerebral haemorrhage [87].

TMEM63A is an osmosensitive and mechanosensitive calcium-permeable channel expressed by OLs that is important for transducing myelin sheath tension during axonal wrapping and myelin compaction. The key role of this channel in myelination is proved by the evidence that mutations in TMEM63A cause a hypomyelinating LD. In the following section, the role of this channel will be addressed in greater detail.

Finally, the expression of the gap junction proteins connexins (Cxs) increases during OLs maturation [88]. Notably, Cx47, regulated by the OL-specific Sox10 transcription factor, rises in mature Ols, while it is almost absent in OPCs [89]. This expression pattern enables the formation of heterotypic gap junctions between astrocytes expressing Cx43 and myelinating OLs expressing Cx47. The resulting glial syncytium and functional coupling between these cells are essential for maintaining ionic and metabolic homeostasis and support proper myelin formation and function [90]. Noteworthy, mutations in *GJA12*, the gene encoding Cx47, cause PMD-like LD characterized by severe CNS dysmyelination [91].

Moreover, within myelin sheaths, Cx32-homotypic gap junctions between adjacent myelin lamellae, facilitate the diffusion of ions and small metabolites across the myelin layers, particularly in the PNS but also in the CNS. This intramyelinic coupling is crucial for the metabolic support of the periaxonal space and myelin integrity. Mutations in the Cx32 protein lead to Charcot-Marie-Tooth disease, a demyelinating neuropathy [92]. Notably, mice double KO for Cx43 and Cx32 manifest a severe CNS demyelination, with formation of vacuoles in the WM, intramyelinic oedema, axonal degeneration and loss of OLs, unequivocally demonstrating that oligodendroglial/astroglial Cxs are essential for the formation/maintenance of functional myelin [93].

Figure 2 summarizes the ion channel dynamics in OPC and OLs, also including astrocyte contribution described in the following sections.

### 4.3. Astrocytes and Their Ion Channels in Myelin Formation and Stability

Despite the primary role of OLs in myelination, astrocytes also contribute substantially to the process. They originate from radial glia, in the subventricular zone. During development, progenitors undergo a gliogenic switch that gives rise to both astrocytes and OPCs [37,94]. Because astrocytes appear before OPCs, they create a trophic and regulatory environment essential for OPC colonization of the cortex and the onset of postnatal myelination. For instance, astrocytes secrete Platelet-Derived Growth Factor (PDGF) and Fibroblast Growth Factor (FGF), which promote OPC proliferation [95], and release lipids and cholesterol, largely produced by astrocytes themselves, needed for myelin synthesis [96]. Moreover, their ability to maintain ion homeostasis directly influences neuronal activity, axonal health and OLs maturation.

Thus, astrocyte dysfunction could impair OPC maturation and myelin formation, as demonstrated by the discovery of LDs caused by mutations in astrocyte-specific genes [9].

#### *4.3.1.* *The Role of Astrocyte Ion Channels in Integrating Neural Activity to Regulate OPC Differentiation*

**Figure 3:**
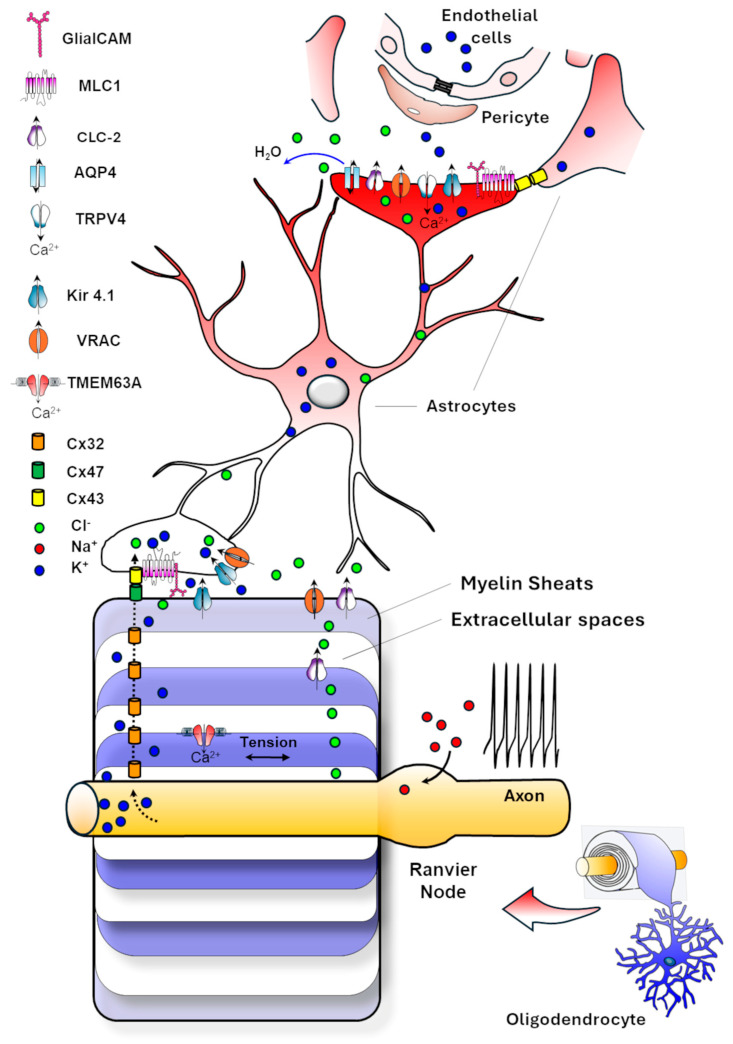
***Molecular mechanisms regulating ion–water homeostasis and cell volume in perimyelinic and perivascular areas of the CNS that are implicated in the pathogenesis of specific LDs***. This figure illustrates the interplay between astrocytes, oligodendrocytes (OLs) and neurons in supporting electrolytes and water homeostasis along the perimyelinic and perivascular spaces. Alterations in these processes are implicated in the pathogenesis of some LDs. Astrocytes contact vessels forming the BBB (including the basal lamina, endothelial cells and pericytes) with their endfeet and other glial cells (OLs and other astrocytes) via gap junctions forming a functional syncytium that buffers extracellular K^+^ and Cl^−^, and water transients essential for myelin integrity and ion homeostasis. Key molecules involved include: AQP4 that mediates bidirectional water flux along osmotic gradients; ClC-2 that supports Cl^−^ homeostasis and osmotic regulation maintaining myelin ion balance; Cx43, Cx32 and Cx47 that form gap junctions for diffusion of electrolytes and metabolites, and facilitating the spatial K^+^ buffering; GlialCAM that stabilizes MLC1 and ClC-2 at the plasma membranes enhancing ClC-2 current; Kir4.1 that coupled to AQP4 coordinates ions and water movements and reuptake/release of extracellular K^+^; MLC1 expressed at astrocyte junctions and perivascular endfeet that regulates VRAC and TRPV4 function, promotes Kir4.1 expression at the plasma membrane, and support ion fluxes in response to cell swelling; TMEM63A that contributes to mechanical signal transduction in astrocyte and OLs in response to cell swelling and membrane tension; TRPV4 that modulates VRAC and AQP4 activity in response to changes in extracellular osmolarity and mechanical stress; VRAC that restore cell volume after osmotic stress by a mechanism called Regulatory Volume Decrease (RVD).

Astrocytes integrate neuronal electrical activity via ion channels to regulate OPC differentiation. In vitro mixed-cultures of OPCs, astrocytes, and neurons stimulated with 10 Hz bursts show a threefold increase in myelination compared with unstimulated or TTX-treated cultures [97]. This effect is mediated by purinergic P2X receptors, ATP-gated channels permeable to Na^+^, K^+^, and Ca^2+^, promoting the release of leukaemia inhibitory factor (LIF) from astrocytes. LIF acts on OPCs to enhance their differentiation and myelination. Astrocytes also decode neuronal activity with intracellular Ca^2+^ signalling. This Ca^2+^ influx, activated by neuronal firing through Cav1.2 and P2X receptors, triggers in astrocytes a paracrine release of gliotransmitters, as ATP, glutamate, and cytokines, facilitating OPC development [92]. Astrocytic mechanosensitive cation channels, such as Piezo1 [79,98], expressed in the astrocytes’ exploratory processes, sense the extracellular matrix environment, further linking neuronal activity and vascular dynamics to myelination. This channel responds to the osmotic or mechanical stress generated by neurovascular coupling. Activation of Piezo1 evokes intracellular Ca^2+^ transients in primary cultured astrocytes and promotes gliotransmitters release [78], producing slow inward currents flowing into neighbouring neurons through NMDARs [99]. Piezo1 deletion in mouse astrocytes impairs neurogenesis, cognitive behaviours, long-term potentiation, and learning, and reduces the overall hippocampal volume. On the contrary, Piezo1 overexpression in mice enhances mechanotransduction and improves neurogenesis and cognitive tasks [78]. Moreover, astrocytes form extensive astrocyte-oligodendrocyte gap junction networks through Cx30 and Cx43, enabling K^+^ siphoning and metabolic exchange with oligodendrocytic Cx47 (Figure 3). This astrocyte-oligodendrocyte coupling is crucial for maintaining myelin homeostasis and protecting myelin during high-frequency neuronal activity [100]. Cx30/Cx47 double-deficient mice revealed severe vacuolization and myelination defects in all WM tracts of the CNS associated with fewer OL development [93].

#### *4.3.2.* *Astrocyte-Mediated Regulation of Ion–Water Homeostasis in Myelination*

Astrocytes are electrically non-excitable and maintain a hyperpolarized resting membrane potential near –80 mV. This property depends primarily on the inward-rectifying K^+^ channel Kir4.1 and the Na^+^/K^+^-ATPase, which together also enable spatial buffering of extracellular K^+^ released during neuronal firing [101,102]. Kir4.1 is enriched in astrocytic endfeet around blood vessels forming the BBB and nodes of Ranvier, where it helps to stabilize ionic gradients critical for axonal conduction and long-term myelin maintenance. In humans, mutations in Kir4.1 cause the EAST/SeSAME syndrome, a rare channelopathy characterized by epilepsy, ataxia, sensorineural deafness, and tubulopathy, and where myelin vacuolation and intramyelinic edema have been reported by neuroimaging [103]. These findings are in accordance with the observation of massive spongiform vacuolization of the WM with demyelination and axonal, coupled to metabolic defects in Kir4.1^−/−^ mice [39,101,104], underscoring the significance of these channels and the correct K^+^ buffering for myelin integrity [105].

However, the astrocyte-mediated maintenance of ionic homeostasis in the brain tissue involves a variety of ion channels and associated modulators. As illustrated in Figure 3, astrocytes, by contacting blood vessels with their endfeet from one side and myelinating oligodendrocytes and other astrocytes via gap junctions on the other, coordinate the buffering of extracellular K^+^ and Cl^−^ ions and other osmolytes in response to neuronal activity, promoting their release into the blood circulation along with water. Alongside Kir4.1, other key molecules involved in this process include: the water channel AQP4, mediating the bidirectional water flux along osmotic gradients; Connexins 43, 32 and 47 which form gap junctions allowing the diffusion of electrolytes and metabolites between OLs and astrocytes as well as astrocyte themselves; the ion channel ClC-2 and its modulator GlialCAM which support Cl^−^ and water fluxes; the TMEM63 channel contributing to mechanosensitive signaling in astrocytes and OLs in response to swelling and membrane tension; the calcium channel TRPV4 favouring AQP4 function and water exchanges; the membrane protein MLC1 which binds and regulates membrane expression of GlialCAM, ClC-2 and of the volume regulated anion channels VRAC.

VRACs are anion channels predominantly expressed in astrocytes and neurons, where they contribute to cell volume and ionic homeostasis regulation. By opening in response to cell swelling and mediating the efflux of Cl^−^ and other anions, small organic osmolytes and neurotrasmitters (I^−^, Br^−^, taurine, glycine, glutamate, aspartate), they restore cell volume through regulatory volume decrease (RVD) [106]. Notably, reduced VRAC function in astrocytes caused by mutations in MLC1 contributes to the pathogenesis of the vacuolating LD MLC (see sections below). Similarly, mutations in the Cl^−^ channels ClC-2 and its modulator GlialCAM, as well as in the water channel AQP4, proteins abundantly expressed at astrocyte endfeet contacting the BBB, are responsible for dysmyelinating LDs (see the specific sections below).

**Figure 4:**
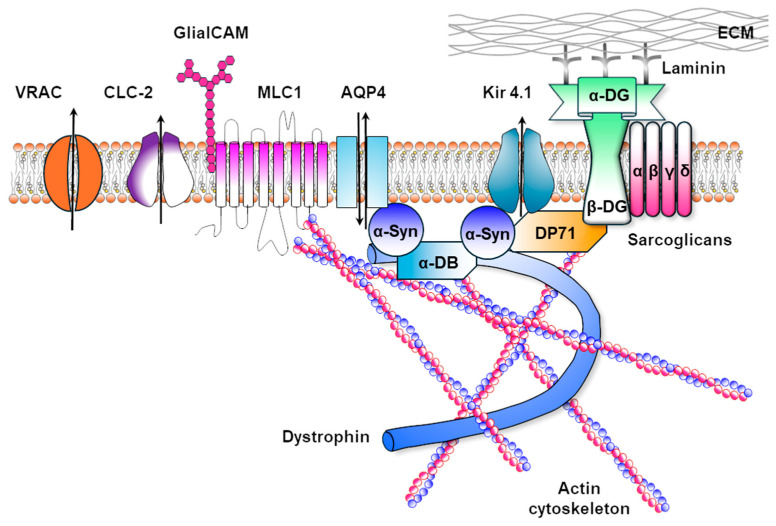
***The multiprotein complex controlling ion and water homeostasis at the astrocytic endfeet. All the molecular components of this complex have been implicated in the pathogenesis of distinct LDs***. This figure depicts the organization of the protein complex constituted by MLC1-GlialCAM-ClC-2-Kir4.1-AQP4 and VRAC at the astrocytic endfeet contacting the blood-brain-barrier. The anchorages to the extracellular matrix and to the internal cytoskeleton are provided by the dystrophin-dystroglycan complex (DGC). DG: dystroglycan, DB: dystrobrevin, DP71: Dystrophin short product 71 kDa, Syn: syntrophin, ECM: extracellular matrix.

The identification of LD-causing mutations in proteins and ion channels functionally and structurally linked at the astrocyte endfeet contacting the BBB and that are involved in ion and water exchanges strongly suggests that proper astrocyte-mediated control of ionic homeostasis at this site is crucial for myelin formation and maintenance. Figure 4 depicts the astrocytic endfoot-associated complex, including MLC1-GlialCAM-ClC-2-Kir4.1-AQP4 and VRAC. This protein assembly is stabilized through binding to the dystrophin-dystroglycan complex (DGC), which connects the cytoskeleton to the extracellular matrix [107].

This ensemble of ion channels and their associated modulators participate in ion/water exchanges between the parenchyma and the blood circulation, as well as in volume change regulation, also in dependence of cytoskeleton/extracellular matrix signalling, ensuring the dynamic regulation of ions and water necessary for proper myelin function, neuronal signalling, and overall CNS homeostasis.

#### *4.3.3.* *The Myelination Energy Costs Are Supported by Astrocytes*

Due to the high energy costs required for myelination, a constant supply of glucose must be taken up from the blood flow by perivascular astrocytic endfeet enriched in GLUT-1, which is subsequently converted into glycogen as a stored form by astrocytes [108]. These glycogen stores can sustain OPC myelination through the lactate shuttle across the astrocyte membrane through monocarboxylate transporters MCT4, allowing lactate to pass in cotransport with protons [65]. Lactate is then retrieved by myelinating OLs through the MCT1 transporter [109].

Energy deprivation can cause a delay in myelination, or dysmyelination, both in culture systems [110] and in in vivo models [111].

In summary, astrocytes possess the ability to store glycogen and lactate, which can be mobilized during periods of heightened neuronal activity, providing crucial metabolic support not only to neurons but also to myelinating oligodendrocytes [112,113,114].

### 4.4. Microglia and Myelin

Microglial cells are the resident immune population of the CNS, accounting for about 5–10% of all cells. They form a heterogeneous population which takes part in normal brain development, homeostasis and maintenance of neuronal function by modulating neurogenesis, neuronal survival, and synaptic plasticity. Microglia role in CNS development is mainly related to their capacity to eliminate unnecessary structures (i.e., synaptic pruning and myelin debris removal) and to secrete trophic factors [112,113,114,115]. The neuroprotective and regenerating functions of these cells throughout life are mediated by their capacity to support oligodendrocyte development and function and myelin formation/preservation in response to neuronal activity and environmental signals [114,116,117]. Very recent studies employing dynamic imaging techniques and animal models of neurological diseases, such as multiple sclerosis and spinal cord injury, have proved that microglia exert a neuroprotective role, also by establishing direct contacts with neurons at Ranvier Nodes. These studies identified this site as a major hub for microglia-neuron communication that mediates their promyelinating effect following injury [118,119]. In response to neuronal activity and damaging signals associated with the release of K^+^ and ATP, microglia wrap around axons at Ranvier Nodes to prevent axonal degeneration from progressing beyond the nodes, a process involving the microglial two-pore domain channel THIK-1 and the purinergic P2Y12 receptors [118,119]. Evidence that microglial interaction at nodes increases during remyelination after injury compared with healthy myelinated tissue further support the role of microglia in promoting efficient repair.

Interestingly, the pharmacological blockade or genetic ablation of Colony Stimulating Factor 1 Receptor (CSF1R), a microglial tyrosine kinase receptor essential for microglial development and survival, alters oligodendrocyte maturation and myelin structure, confirming that the function of these glial cells is essential for proper myelin development and maintenance [120,121]. A significant confirmation of this evidence came from the discovery that loss-of-function mutations in the CSF1R gene cause hereditary diffuse leukoencephalopathy with axonal spheroids (HDLS), an adult-onset leukodystrophy where myelin degeneration occurs, associated with axonal damage [122,123]. 

Although microglia express a large number of ion channels, in normal and activated conditions [14,124], their specific involvement in myelination is only recently emerging. Microglial ion channels whose activation negatively affects myelin formation in mouse models of demyelination include the mechanosensitive channel Piezo1 [125], the voltage-gated proton channel Hv1 [117,126,127,128], and the cation channels TRPA1 [129], mainly by mechanisms involving oxidative and inflammatory stress-induced injury to myelin or OPCs/OLs.

Specifically, although the negative regulation of myelin formation induced by Piezo1 is mainly due to its role in OPCs and OLs, microglial cells also express these mechanochannels. Activation of Piezo1 in microglia contributes to demyelination through the secretion of inflammatory cytokines and chemokines (CCL25 and IL18), which in turn induces ferroptosis in OLs [125].

The voltage-gated proton channels Hv1 are expressed on the plasma membrane and phagosomes in many different cell types and tissues. They open at relatively positive transmembrane voltages when the electrochemical driving force is directed outward, thereby generating an outward H^+^ current to extrude acid. In the CNS, the Hv1 channels are selectively expressed in immune cells, mainly dendritic cells and microglia [126]. Studies in Hv1 KO in vitro and in vivo models, either in basal conditions or following various damaging stimuli, revealed that Hv1 plays a significant role in neuroinflammation, oxidative stress, and mitochondrial dysfunction associated with neurological diseases. The lack of this channel decreases ROS and inflammatory cytokine production, protecting the brain from oxidative stress, demyelination, traumatic injury and ageing, and favours OPC differentiation and the polarization of microglial cells toward a protective phenotype [127,128,130,131].

Cuprizone-induced demyelination in TRPA1 KO mice revealed significantly attenuated demyelination in these animals compared to controls. Histological analysis of brain tissue showed a reduced accumulation of microglia and activated astrocytes in demyelinated areas [129]. On the contrary, studies on TRPV1 KO mice proved that the activation of these cation channels supports myelin repair following cuprizone-induced demyelination via a positive regulation of microglial function. In particular, TRPV1 expression and activation facilitate myelin repair by favouring the recruitment of microglial cells to the lesioned areas and their phagocytic function, thus potentiating myelin debris clearance [132]. Noteworthy, pharmacological activation of the ATP-sensitive potassium (KATP) channels expressed by microglial cells can provide neuroprotective and anti-inflammatory effects, reducing demyelination in the mouse models of multiple sclerosis by inhibiting the release of inflammatory cytokines [133,134].

## 5. Leukodystrophy Classification: Pathological Mechanisms and Glial Cell Contribution

The increasing discoveries revealing the genetic basis of LD and the advancements in the comprehension of the complex cellular relationships supporting the synthesis and maintenance of healthy WM, prompted a classification of LD based on the primary cellular structure affected in the pathological process [3,4]. This classification encompasses “myelin disorders”, which develop primarily due to oligodendrocyte or myelin defects; “astrocytopathies”, indicating disorders characterized by primary astrocyte dysfunction; “leuko-encephalopathies”, or “axonopathies” developing from neuronal or axonal dysfunction; “microgliopathy” related to defects in microglial cell genes and “leuko-vasculopathies”, for vascular pathologies [3].

More recently, evidence showing that LDs can be caused by defects in proteins that regulate diverse molecular processes and intracellular organelle functions prompted an additional classification based on the defective cellular processes or cellular structures involved in specific LD pathogenesis [135]. This latter classification includes: lysosomal disorders; peroxisomal disorders; metabolic disorders caused by defects in amino acid and organic acid metabolism; mitochondrial disorders; DNA repair disorders involving abnormalities in DNA repair mechanisms; protein translation defects causing alteration of protein synthesis; defects in ion and water homeostasis; myelin protein disorders, genetic vasculopathies [2,3]; finally, defects in mechanosensitivity of plasmalemma and cytoskeletal structures have also been identified as cause of LDs.

It is worth noting that these classification systems have their limitations, as some LDs can involve multiple cell types or pathways, and the underlying mechanisms are complex and often still elusive. In many of these cellular compartments and biological processes, ion channels are involved and directly or indirectly linked to disease pathomechanisms, as will be addressed in the next sections.

## 6. Direct Involvement of Glial Ion Channels in LD Pathogenesis

While traditionally associated with metabolic enzyme defects or myelin protein mutations, there is increasing evidence that alterations of ion channels function also play a role in the abnormal development or destruction of myelin sheaths during LDs. Indeed, mutations in genes encoding ion channels have been described in two LDs, the hypomyelinating HLD19 LD and the ClC-2 LD, also termed *CLCN2*-related leukoencephalopathy, caused by mutations in the *TMEM63A* and *CLCN2* genes, respectively.

### 6.1. Mutations in the Mechanosensitive Channel TMEM63A Cause HLD19 Leukodystrophy

#### 6.1.1 TMEM63A

TMEM63A belongs to a family of channels which includes the two paralogues TMEM63B and TMEM63C. These latter are homologous to the OSCA1.1/1.2 channels found in plants and known as osmosensitive and mechanosensitive calcium-permeable channels [136,137].

Mechanosensitive channels are ion channels activated by mechanical forces, which convert the physical forces into electrical and biochemical signals to stimulate a variety of intracellular responses and regulate important physiological functions.

In mammals, the mechanosensitive channels include Piezo1 and 2 channels, the K2P two-pore domain potassium channels, and the TMC1/2 channels [138,139]. These are involved in specific physiological functions such as touch, breathing, blood pressure regulation, proprioception, hearing, itch sensation, and resting membrane potential modulation. However, the largest family of eukaryotic mechanosensitive channels is represented by the OSCA expressed in plants and TMEM63 proteins, including the TMEM63A, B, and C found in animals [136].

TMEM63A is expressed in the brain, mainly in OLs but also in astrocytes, kidney, lung, pancreas, and other tissues; TMEM63B is also found in neurons with a possible role in neurodevelopment, hearing and osmosensation. *TMEM63C* is less expressed compared to TMEM63A/B and is highly enriched in neurons [140]. Structurally, TMEM63 channels are preferentially organized into monomers, in contrast with the dimeric/multimeric assembly of OSCA proteins, Piezo and TMC channels.

The heterologous expression of TMEM channels in Human Embryonic Kidney (HEK) cells showed stretch-activated currents and suggested roles in volume regulation and mechanosensory responses [81,82,140]. It has been proposed that TMEM63 evolved to function as a high-threshold mechanosensitive ion channel, complementing the low-threshold activation of Piezo and TMC1 channels [141].

TMEM63A currents are force-activated and voltage-insensitive, with a high threshold for pressure activation with slow activation and deactivation. The pressure-dependent TMEM63A currents in cell membrane patches were found to correlate with cell size. Moreover, TMEM63A activation would cause an inward Ca^2+^ current, plasma membrane depolarization, and a sustained [Ca^2+^]_i_ increase in response to high stretch [82]. Interestingly, very recent papers demonstrated additional lipid scramblase activity of these channels [142,143,144]. Their conserved TM4 hydrophobic gate has the potential for phospholipid permeation [142]. The asymmetric distribution of phospholipids between the outer-(phosphatidylcholine, sphingomyelin, and glycosphingolipids enriched) and the inner-leaflet (phosphatidylethanolamine, phosphatidylserine, and phosphatidylinositol enriched) [26,145,146], could influence the adsorption of intrinsically disordered myelin proteins, such as MBP, in the plasma membrane, and thus affect myelin compaction [147]. Therefore, any mechanism affecting lipid scrambling might result in myelin dysfunction.

#### 6.1.2 TMEM63A and LD Pathogenesis

The involvement of TMEM63A channels in LD pathogenesis was first proved when heterozygous missense mutations in *TMEM63A* gene were found in four children affected by HLD19. This is an LD characterized by hypomyelination in the corpus callosum and deep subcortical WM, nystagmus, ataxia, motor developmental delays, and seizures, resembling PMD but with an unusual remitting phenotype [10]. Cellular models obtained by transfection of TMEM pathological variants into Piezo1-KO HEK cells revealed that these mutations were loss-of-function, being the mutant cells unable to induce stretch-activated currents in response to mechanical stimulation [10]. More recently, two additional LD patients carrying 2 of the already described mutations were found [148], followed by the identification of new mutations (4 missense and a null mutation) in patients with classical or more severe phenotypes [35,149,150,151,152] and with an unusual adult-onset [153]. To study the molecular mechanism of TMEM63-associated brain dysfunctions, Wang and collaborators generated Tmem63a EGFP knock-in and Tmem63a^−/−^ mice. These models allowed them to reveal that TMEM63A is abundantly expressed in OL lineage. TMEM63 KO mice exhibited temporary hypomyelination at ~2 weeks of age, with a reduced number of myelinated axons and thinner myelin sheet deposition associated with oligodendrocyte differentiation defects that recovered between P21 and P28 [35].

**Figure 5:**
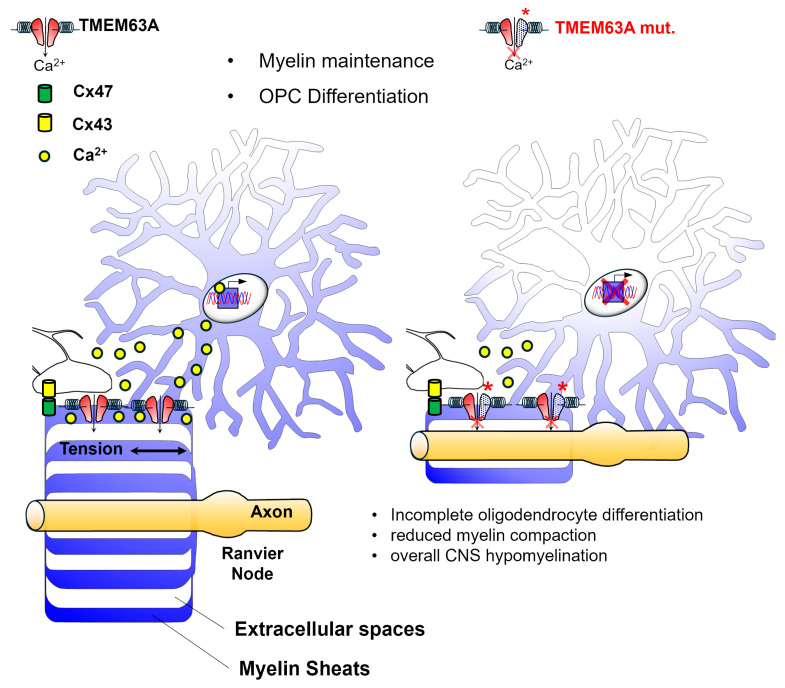
***Proposed Molecular Mechanism of the Hypomyelinating Leukodystrophy HLD19***. The mechanosensitive TMEM63A ion channels, which are located at the plasma membrane and lysosomal membranes of oligodendrocytes (OLs), physiologically respond to tensions caused by OL membrane wrapping and twisting around the axon, as well as osmolar stress from axon-oligodendrocyte interactions, during intense firing. TMEM63A activation allows intracellular Ca^2+^ entry, which in turn induces OL maturation, thus promoting the proper myelin sheath formation around axons. In HLD19, pathogenic TMEM63A variants (indicated by asterisks) decrease translocation to the plasma membrane or reduce channel function, leading to defective Ca^2+^ entry and OL maturation. This results in incomplete oligodendrocyte differentiation, reduced myelin compaction, and overall CNS hypomyelination.

These observations revealed that *TMEM63A* KO mice recapitulate the improving phenotype of human patients. Interestingly, the defects in OL differentiation observed in the brain tissue of *TMEM63A* KO mice were further confirmed in primary cultures of OPCs, where the induction of OL differentiative stimuli causes a temporary deficiency of differentiation of KO OLs that is rescued by virus-based re-expression of the WT, but not mutated, TMEM63A [35]. These results suggest that TMEM63A regulates myelination in a cell-autonomous manner. The same authors also showed that Ca^2+^ entry through TMEM63A channels in OLs in response to mechanical stimulation significantly contributes to the Ca^2+^ signal at the early stage of OL differentiation, highlighting a critical role for TMEM63A-mediated Ca^2+^ influx in OL differentiation and providing insights into the mechanics for *TMEM63A* mutation-related hypomyelinating diseases (Figure 5).

In another very recent study, using cellular models, the authors confirmed that pathological mutations affect protein trafficking at the plasma membrane and at lysosomes, two intracellular compartments where TMEM63A has been previously localized [154]. These findings suggest that TMEM63A channels most likely modulate myelination by acting at the OL surface but not excluding a contribution of mechanosensitive processes within subcellular organelles. Most importantly, loss of TMEM63 function results in a transient reduction in myelin in both mice and zebrafish, demonstrating an evolutionary conservation of this channel. A detailed analysis of TMEM63A expression confirmed its specificity to the OL lineage; however, in contrast to observations by Wang and collaborators, no defects in OL maturation were reported. Differences in mouse strains used across studies may account for these discrepancies. Despite some inconsistencies, these models offer important insights into the molecular mechanisms underlying the HLD19.

The identification of TMEM63A as a key regulator of myelination opens avenues for therapeutic research, not just for HLD19 but possibly for other myelin disorders.

### 6.2. ClC-2 Mutations Cause a Vacuolating Leukodystrophy

#### *6.2.1.* *ClC-2*

ClC-2 is a voltage-gated chloride channel widely expressed in mammalian tissues. It belongs to the CLC family, which includes nine mammalian homologs functioning as Cl^−^-selective channels and Cl^−^/H^+^ transporters that are expressed at the plasma membrane or in intracellular organelles [155,156,157]. ClC-2 is activated by membrane hyperpolarization, leading to inward-rectifying currents. The current develops gradually and deactivates slowly upon depolarization. Typically, both activation and deactivation occur slowly compared to other ion channels. ClC-2 shows the typical anion permeability sequence of the CLC channels: Cl^−^ > Br^−^ > I^−^ > F^−^. The voltage-dependent gating is modulated by extracellular pH and intracellular Cl^−^ concentrations, with higher H^+^ concentration facilitating activation. Intracellular Cl^−^ itself can also stabilize the open state of the channel. ClC-2 is found in nearly all organs, including the brain, heart, intestines, and lungs, where it plays essential roles in electrogenesis, cell volume regulation, and the maintenance of ion gradients. Within the CNS, ClC-2 is expressed in both neurons and glial cells. Like the other CLC channels, ClC-2 proteins adopt a homodimeric structure in which each monomer forms an identical and independent ion-conducting pore. Each monomer is composed of 18 transmembrane helices, with cytosolic N and C-terminal domains. Functional mutational analysis revealed some structural requirements for activation, with the intracellular N-terminus responsible for voltage- and pH-sensitive gating, and the C-terminal holding two conserved cystathionine-β-synthase (CBS) domains critically modulating channel intracellular trafficking [12,155,157]. It is also likely that this latter region is involved in gating regulation since its truncation or mutation leads to faster channel activation/deactivation. ClC-2 also showed osmotic sensitivity, being activated by cell swelling, such as during hypotonic stress, linking it to volume regulation functions in cells.

#### *6.2.2.* *CLC-2 and LD Pathogenesis*

In humans, mutations in the *ClC-2* gene have been associated with ClC-2 LD, also named *CLCN2*-related leukoencephalopathy. This disease, caused by loss-of-function mutations in the *CLCN2* gene, leads to myelin vacuolation and WM oedema, with a progressive degeneration [11]. Affected patients manifest symptoms like ataxia, spasticity, seizures, nystagmus, and cognitive decline [11,158] often associated with male infertility and visual impairment. A similar phenotype has been observed in *ClC-2* KO mice, which displays severe retinal degeneration leading to blindness, infertility, and prominent vacuolization of myelin in the brain tissue [156,159].

**Figure 6:**
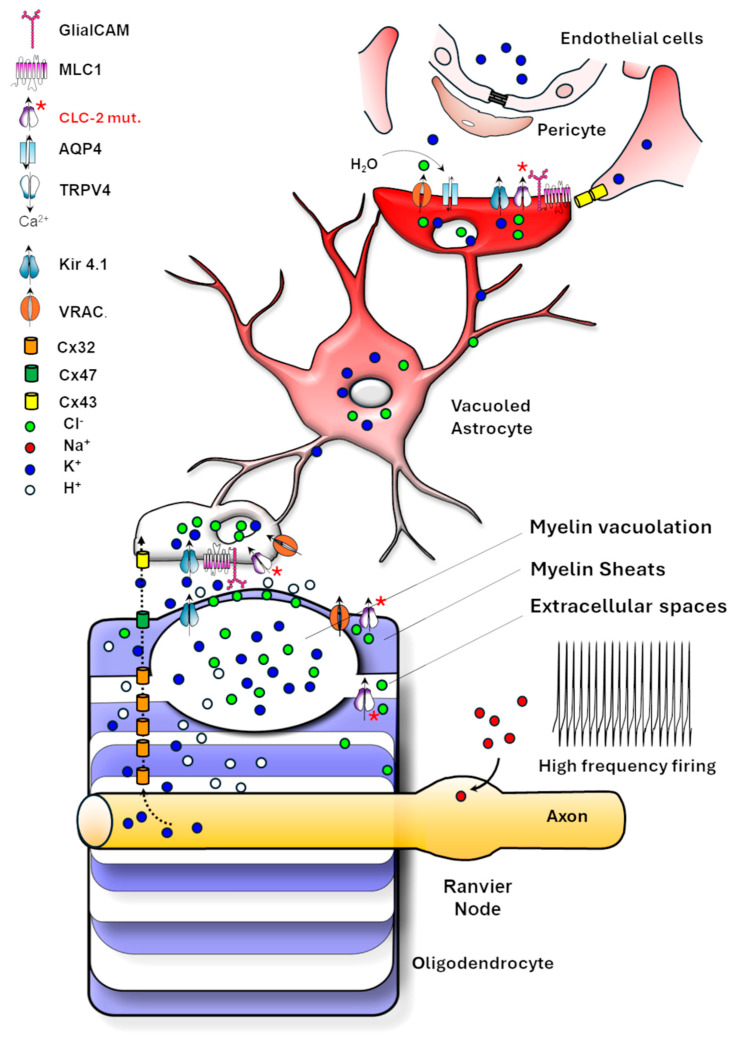
***Proposed Molecular Mechanism of CLCN2-related Leukoencephalopathy***. The voltage-gated, Cl^−^-permeable, ClC-2 channel expressed in astrocytes and oligodendrocytes contributes to membrane potential maintenance, cell volume control, and Cl^−^ flux regulation in response to osmotic changes. In astrocytes, ClC-2 is concentrated in perivascular and perisomat-ic membranes, where it interacts with GlialCAM. This interaction promotes its correct localiza-tion and membrane stability. Mutations in CLCN2 (indicated by asterisks) result in loss of func-tion of the ClC-2 channel, with reduced Cl^−^ efflux and consequent alteration of osmotic balance leading to intracellular accumulation of ions and water, astrocytic oedema and white matter vacuolation, possibly due to K^+^ and Cl^−^ accumulation in the more external perimyelinic spaces.

To date, the precise pathophysiology of this LD is uncertain, but the central role played by both OLs and astrocytes is emerging. ClC-2 is particularly expressed in these glial cell populations, and its abundance and subcellular localization depend on the physical binding to the GlialCAM protein. This cell adhesion molecule localizes ClC-2 to cell–cell contacts and modifies its functional properties by changing channel gating [160,161]. GlialCAM also directly interacts with the astrocytic MLC1 protein, whose mutations account for the vacuolating LD MLC (see section below) [162,163]. Cellular expression systems and KO mouse models reported that GlialCAM, MLC1 and ClC-2 form a complex enriched at the astrocyte-oligodendrocyte contacts and at astrocyte endfeet contacting the blood vessels [163]. At this site, they colocalize with Kir4.1 and AQP4, two proteins involved in K^+^ ion and water siphoning in the blood circulation (Figure 4). This localization and the overlapping phenotypes of Kir4.1, ClC-2, MLC1 and GlialCAM KO mice showing myelin vacuolation and brain oedema suggest that these proteins take part in the same physiological process [156,159]. The main hypothesis is that defects of the K^+^ ions buffering and water exchanges in response to physiological neuronal activity may be the common pathomechanisms of these myelin diseases. Indeed, Kir4.1, expressed in astrocytes and OLs together with the water channel AQP4, plays a key role in balancing K^+^ and water exchange between glial cells and the vasculature at astrocytic endfeet. In this setting, ClC-2 is thought to provide a charge-balancing current that supports the electrogenic flux of K^+^ into and out of glial cells via Kir4.1 channels. Functional alterations of these proteins, along with GlialCAM that correctly localizes ClC-2 in astrocytes and OLs, can lead to abnormal water and K^+^ accumulation in the brain tissue with consequences on myelin formation and compaction and brain oedema (Figure 6). In addition, the chloride channel ClC-2 expressed in glial cells has been reported to directly affect oligodendrocyte/myelin physiology. Hou and collaborators provided evidence that ClC-2 may be a positive regulator of OPC differentiation and able to contribute to myelin formation and repair [44]. In a very recent paper, it has been proved that dysfunctional ClC-2 in astrocytes derived from both mouse models and human inducible pluripotent stem cells (iPSC) impairs the development of the OL lineage in vitro and delays remyelination in vivo through the upregulation of osteopontin (SPP1), a cytokine-like inflammatory molecule, from diseased astrocytes [164].

Overall, these findings highlight the need for a glial-mediated effective and precise regulation of ionic balance and chloride and water homeostasis for proper oligodendrocyte development and myelin formation and maintenance.

## 7. Indirect Involvement of Ion Channels in LD Pathogenesis

Although the ion channels mentioned above play a primary role in the pathogenesis of some LDs, other ion channels also contribute significantly to the pathological mechanisms causing myelin dysfunction in these diseases, even if they are not the direct targets of genetic mutations, as seen in megalencephalic leukoencephalopathy with subcortical cysts (MLC).

### 7.1. MLC1 Protein: An Ion Channel Modulator 

MLC1 is a 377 aa membrane protein which shows no similarities with other known proteins, except for a very low sequence homology with a subunit of the ion channel Kv1.1. For this reason, and for its involvement in the regulation of ion and water balance in astrocytes reported in different in vivo and in vitro models, it has been considered a putative ion channel [165,166]. However, to date, the proper function of MLC1 is still elusive. Integrated computational approaches suggest MLC1 as a highly hydrophobic membrane protein, composed of 8 transmembrane domains which form 2 specular 4-domain structures linked by an intracellular aminoacidic loop and expose the N and C terminal into the intracellular side. Biochemical studies also revealed that MLC1 is phosphorylated by PKC, PKA and CAMKII kinases and that it is a calcium/calmodulin binding protein whose localization and function depend on the intracellular calcium concentrations and calcium release from endoplasmic reticulum stores [167,168,169].

### 7.2. MLC1 and LD Pathogenesis

The discovery of the MLC1 protein originated from the identification of patients affected by the same LD, megalencephalic leukoencephalopathy with subcortical cysts (MLC disease), who carried mutations in the same gene, subsequently named *MLC1* [170,171]. Mutations in *MLC1*, with a recessive way of transmission, have been found in 80% of patients affected by MLC. Later, dominant and recessive mutations in a second gene encoding the adhesion molecule GlialCAM which forms a protein complex with MLC1, have been found in 10–15% of MLC patients without mutations in the *MLC1* gene [172]. More recently, mutations in the G-protein-coupled receptor *GPRC5B* gene and the astrocytic water channel *AQP4* gene have been reported in a few MLC patients [173].

In the brain, MLC1 is specifically expressed by astrocytes and particularly localized at perivascular endfeet and subpial astrocytic processes [174,175,176]. The exclusive astrocytic expression of the MLC1 protein revealed that in MLC disease myelin degeneration is secondary due to a primary astrocytic dysfunction, classifying MLC as an astrocytopathy. MLC disease usually occurs during infancy with macrocephaly manifesting some months after birth, followed by slow, progressive motor deterioration and seizures. MRI consistently shows diffuse WM oedema and distinctive subcortical cysts [148]. Histochemical analysis of brain tissue samples revealed the presence of vacuoles in the external layers of the myelin sheets, indicative of myelin compaction defects [148].

**Figure 7:**
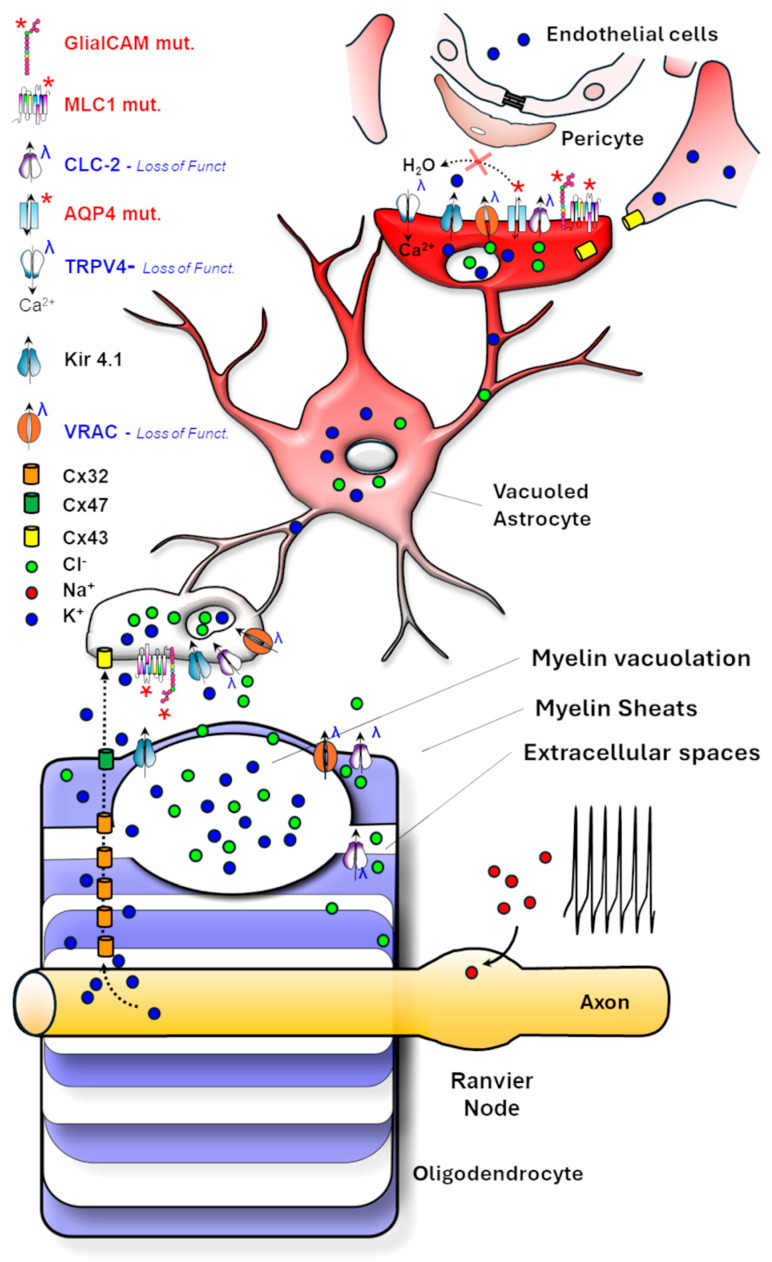
***Proposed Molecular Mechanism of MLC Leukodystrophy***. The MLC1–GlialCAM complex, located at the astrocytic membrane, interacts with the VRAC and ClC-2 channels to regulate anion flux and osmotic homeostasis. In synergy with AQP4 and Kir4.1, which control water and potassium movements, and with TRPV4, a sensor of cellular swelling, these complexes maintain volume and ionic balance. Mutations in MLC1 or GLIALCAM (indicated by asterisks) compromise these mechanisms, causing astrocytic edema and white matter vacuolation, possibly due to K^+^ and Cl^−^ accumulation in the more external perimyelinic spaces.

The pathogenesis of MLC centres on defective ion and volume regulation in astrocytes (Figure 7). Indeed, the diffuse WM oedema, fluid-filled cysts, and myelin vacuolation characterizing MLC patients are suggestive of ion/water imbalance defects. Mouse models deficient in *MLC1* or *GlialCAM* partially recapitulate key aspects of human disease, including brain oedema and myelin vacuolation, which, however, is restricted to the cerebellum [163]. Electrophysiological studies in MLC1-deficient astrocytes show reduced volume recovery following osmotic challenges and defects in the activation of hyposmotic-induced chloride currents [163].

### 7.3. The MLC1-Associated Protein Complex

Although MLC1 is not a classical ion channel or transporter, it interacts physically and/or functionally with key regulators of astrocytic volume and synaptic homeostasis, including the inward-rectifying K^+^ channel Kir4.1, the Na, K-ATPase [177], the chloride channel ClC-2 [159], the water channel AQP4 [174,175], the calcium TRPV4 [175,178] and the volume-regulated anion channels VRAC [162], as well as the DGC, the protein complex anchoring most of these channels to the astrocytic plasma membrane (Figure 4) [174,175]. These proteins are essential for K^+^ clearance and volume regulation, and, thus, to keep the ionic gradients and membrane potential necessary for effective myelin formation and compaction and synaptic transmission. Interestingly, mutations in MLC1 cause a decrease in Ca^2+^ influx through TRPV4 channels, in Cl^−^ currents through VRAC and ClC-2 channels, and in Kir4.1 current. Moreover, MLC1 protein also interacts with the vacuolar ATPase at endosomal organelle membranes, where it takes part in the regulation of early endosome acidity, thus influencing cargo protein recycling/degradation, including ion channels and receptors [179]. All these findings suggest that MLC1 acts as an ion channel modulator, with a crucial role in supporting proper ion and water exchanges in the brain. MLC1 functional disruption can lead to abnormal swelling of astrocytes that become incapable of regulating ionic homeostasis during the physiological electrical activity and in response to pathological conditions such as traumas, inflammation, and oxidative stress [169], thus leading to the worsening of patient symptoms. 

In this scenario, MLC1 may be primarily involved in K^+^ buffering along with GlialCAM and ClC-2, similarly to ClC-2-associated LD, with which MLC disease shares several pathological features, including myelin vacuolation, brain oedema, and a similar localization in perivascular astrocyte endfeet (Figure 6 and Figure 7). Accordingly, very recently, we reported Kir4.1 dysfunctions in astrocytes derived from iPSC of MLC patients carrying mutations in the MLC1 protein [180]. Notably, mutations in MLC1 or GlialCAM lead to channel mislocalization and reduce ClC-2 conductance [160], suggesting the involvement of common pathological pathways in these LDs.

MLC LD highlights the significance of astrocytic ion channels in preserving and supporting myelin integrity and compaction, implicating astroglial ionic imbalance as a primary driver of pathology.

Table 1 summarizes the cellular/molecular and clinical features of the LDs described above.

## 8. Conclusions

The studies discussed in this review highlight the crucial and intertwined roles of ion channels expressed by myelin-forming cells (OPCs and OLs), as well as by other glial components such as astrocytes and microglial cells, in both the formation of myelin and the preservation of its functional integrity.

In general, OPCs express a complex repertoire of ion channels that support their migration and proliferation in response to neuron-derived signals, with which they establish bidirectional communication. During oligodendrocyte maturation, a qualitative and quantitative shift in ion channel expression occurs. These changes enable mature OLs to perform more specialized functions, including metabolic support for axons and the maintenance of ionic homeostasis, two essential conditions for proper myelin synthesis and stabilization. In this context, astrocytes and microglia provide essential support for OPC and oligodendrocyte development and maturation, and for myelin structural and functional integrity, through the activation of specific molecular programs that involve ion channel activation or inhibition.

Unravelling the role of specific ion channels expressed by different glial cells can provide valuable insights into the molecular mechanisms underlying demyelinating diseases and help identify potential therapeutic targets for distinct pathological contexts.

LDs, being monogenic demyelinating disorders, represent an important model for such studies. The rapid advances in sequencing and imaging technologies for the identification and characterization of LD patients and their causative gene mutations, together with the development of relevant human and animal models, complemented by multi-omics approaches and functional analyses, have allowed the identification of novel molecular players, including glial ion channels, in myelin biology. This is exemplified by the discovery that mutations in ion channels such as TMEM63A and ClC-2, as well as in the ion channel modulators MLC1/GlialCAM, cause hypomyelinating, and vacuolating LDs. These findings have expanded our understanding of the molecular events underlying healthy myelin development and maintenance and revealed ionic and water homeostasis and mechanosensitivity as crucial mechanisms.

Deepening our knowledge of cellular and molecular events causing myelin defects in LDs can open new avenues for investigating key contributors in other demyelinating diseases, suggesting the great potential of a cross-pathological, systems-level approach to study demyelination mechanisms.

## 9. Future Perspectives

Considering the complexity of myelin biology and the wide range of molecular and cellular elements involved in myelin development, maintenance, and recovery from pathological insults, further investigation is required to identify the key elements that may serve as targets for pharmacological intervention in LDs, with potential cross-applicability to other demyelinating disorders. In this context, specific ion channels emerge as particularly promising targets, including chloride channels (ClC-2, VRAC, and the TMEM16 family), mechanosensitive channels (PIEZO1/2, TRPV4, TMEM63A/B), and axon–OPC activity-dependent Na^+^ and Ca^2+^ channels, all of which warrant dedicated investigation in LD-relevant models.

Of particular interest is the synaptic-like communication between axons and OPCs, mediated in part by ion channels, which link neuronal activity, OPC differentiation and myelin formation. Emerging technologies, such as multielectrode arrays for real-time monitoring of network activity and 3D-culture systems, and BBB in vitro models based on human iPSC, now offer valuable opportunities to study the myelination process in relation to neural circuit dynamics and interactions within the NVU elements. Models incorporating this level of complexity would be particularly relevant in conditions like LDs caused by mutations in genes expressed in glial cells other than myelin-forming cells. This complexity could be approached, for instance, by combining optogenetic activation of axons with readouts of OPC ion fluxes.

Moreover, investigating the role of ion channels in the physiology of intracellular organelles, structures increasingly recognized involved in LD pathogenesis, could be another promising avenue of research.

Importantly, the pharmacological tractability of ion channels represents an exciting translational opportunity. Since ion channels constitute one of the most common classes of FDA-approved drug targets, a repurposing strategy using existing compounds could offer a rapid route toward therapeutic development for LDs. Integrating detailed mechanistic studies with drug screening and repurposing strategies may accelerate the identification of effective interventions to arrest/slow down the degenerative process and hopefully favour brain repair. For instance, testing Piezo1 inhibitors or ClC-2/VRAC modulators in cellular and animal models of specific LDs may reveal novel potential therapeutic approaches, although caution is warranted given the positive role of astrocytic Piezo1 in neurogenesis.

Preclinical approaches targeting specific ion channels such as Kir4.1 and calcium channels have already been investigated in cellular and animal models of demyelinating diseases, including experimental autoimmune encephalomyelitis (EAE), the animal model of multiple sclerosis, and cuprizone-induced demyelination. These studies highlight the critical role of the Kir4.1 channel, which is also involved in the pathological mechanism of some LDs (Figure 6 and Figure 7) and multiple sclerosis and suggest that its pharmacological activation could be a possible strategy to enhance brain repair in demyelinating diseases [183]. Conversely, the inhibition of calcium channels through a combination of three different channel blockers to reduce Ca^2+^-induced oxidative stress in the cuprizone-induced demyelination model has clarified that excessive Ca^2+^ influx contributes to specific pathological events during demyelination processes [184].

Altogether, these lines of research highlight the value of integrating molecular and functional approaches across different demyelinating disorders, with particular attention to ion channel involvement, as a promising strategy to discover shared molecular targets and accelerate the development of effective therapies.

## Figures and Tables

**Table 1:** Summary of key genetic, functional, cellular, clinical, and neuroimaging features of the three leukodystrophies discussed in the text. MRI features are displayed as signal hyperintensities corresponding to alterations in T2-weighted relaxation times, highlighting the distribution and severity of white matter involvement.

Disease	Mutated Genes	Inheritance	Functional Impact	Cellular Effect	Clinical Phenotype	MRI Features
**HLD19**	*TMEM63A*	Autosomal Dominant	*TMEM63A* mechanosensitive ion channel expressed in OLs. Mutations result in loss of function.	Disrupts OL differentiation and myelin production by impairing Ca^2+^ influx.	Motor dysfunction, cognitive impairment. Severe hypomyelination, with a favorable prognosis in the infantile forms. Progressive dementia/ Parkinsonism in the adult forms.	Diffuse hypomyelination (T2 hyperintensity) of the deep and subcortical WM and spinal cord with improvements over time [150,151,152,153].
***CLCN2*** **LD**	*CLCN2*	Autosomal Recessive	ClC-2 chloride channel expressed by astrocytes and OLs. Mutations result in loss of function.	Loss of ionic and water homeostasis in astrocytes and OLs leads to myelin oedema and vacuolization in white matter tracts.	Ataxia, cognitive impairment, visual impairment, male infertility, and headaches. Slow progression.	T2 hyperintensity in long WM tracts, posterior limbs of the internal capsules, midbrain cerebral peduncles, and middle cerebellar peduncles [158,159,160,161,181,182].
**MLC**	*MLC1* (80% of patients) *GlialCAM* *GPRC5B* *AQP4*	Autosomal Recessive Autosomal Recessive and Dominant Autosomal Dominant Autosomal Recessive	MLC1 ion channel modulator expressed in astrocytes. Mutations cause protein degradation Adhesion molecule MLC1-interactor G-protein coupled receptor Water channel	Impairment of ion/water homeostasis leads to brain oedema and myelin vacuolization, astrocyte swelling associated with maturation defects and Kir4.1 impairment.	Infantile-onset macrocephaly; progressive motor disability, ataxia, spasticity, mild cognitive decline, seizures. Slow progression.	WM oedema (T2 hyperintensity) with subcortical cysts and myelin vacuoles, particularly in the anterior temporal regions [165,170,172,173,180].

## Data Availability

No new data were created or analyzed in this study.

## Abbreviations

**AMPAR**	α-amino-3-hydroxy-5-methyl-4-isoxazolepropionic acid receptor
**ANO**	Anoctamin
**AQP4**	Aquaporin channel 4
**BBB**	blood-brain-barrier
**BEST**	Bestrophin
**CaMKII**	Calcium/Calmodulin-dependent protein kinase II
**Cav**	voltage-gated calcium channels
**ClC-2**	Chloride Channel-2
**CNBD**	Cyclic nucleotide-binding domain ion channels
**CNS**	central nervous system
**CREB**	cAMP-response element-binding protein transcription factor
**CSF**	cerebrospinal fluid
**CSF1R**	colony stimulating factor 1 receptor
**Cx**	connexin
**DGC**	Dystrophin-Dystroglycan Complex
**EAAT**	Excitatory Amino Acid Transporter
**EAE**	experimental autoimmune encephalomyelitis
**FGF**	fibroblast growth factor
**GABAA/B R**	gamma-aminobutyric acid receptors
**GLIALCAM**	Glial Cell Adhesion Molecule
**GLUT-1**	Glucose Transporter Type 1
**GPRC5B**	G protein-coupled receptor, class C, group 5, member B
**HCN**	Hyperpolarization-activated Cyclic Nucleotide-gated
**HDLS**	Hereditary Diffuse Leukoencephalopathy with Spheroids
**HEK**	Human Embryonic Kidney cells
**HLD19**	hypomyelinating leukodystrophy 19
**HSCT**	hematopoietic stem cell transplantation
**Hv**	voltage-gated proton channel
**iNOS**	inducible nitric oxide synthase
**iPSC**	inducible pluripotent stem cells
**KATP**	ATP-sensitive potassium channel
**Kir**	inward-rectifier K channels
**Kv**	voltage-gated potassium channel
**LD**	leukodystrophy
**LIF**	leukaemia inhibitor factor
**MBP**	myelin basic protein
**MCT**	Monocarboxylate Transporter
**MDL**	major dense line
**MLC**	Megalencephalic leukodystrophy with subcortical cysts
**MLC1**	Megalencephalic leukodystrophy with subcortical cysts protein-1
**MRI**	magnetic resonance imaging
**Nav**	voltage-gated sodium channels
**NCX**	Sodium-Calcium Exchanger
**NMDA**	N-methyl-D-aspartate
**NVU**	neurovascular unit
**OL**	oligodendrocyte
**OPC**	oligodendrocyte progenitors
**OSCA**	Hyperosmolality-gated calcium-permeable channel
**P2X**	purinergic ligand-gated ion channels
**P2Y**	purinergic receptors
**PDGF**	platelet-derived growth factor
**PKA**	protein kinase A
**PKC**	protein Kinase C
**PLP**	proteolipid protein
**PMD**	Pelizaeus-Merzbacher disease
**PNS**	peripheral nervous system
**RVD**	Regulatory Volume Decrease
**SOX**	Sry-related High-mobility group (HMG) box transcription factor
**SPP1**	osteopontin
**TMC**	two pore cation channels
**TMEM**	Transmembrane Protein
**TRPA**	Transient Receptor Potential Ankyrin channel
**TRPV4**	transient receptor potential vanilloid channel 4
**VGCC**	voltage-gated calcium channels
**VRAC**	volume-regulated anion channels
**WES**	whole exome sequencing
**WGS**	whole genome sequencing
**WM**	white matter
